# Lactulose Ingestion Induces a Rapid Increase in Genus *Bifidobacterium* in Healthy Japanese: A Randomised, Double-Blind, Placebo-Controlled Crossover Trial

**DOI:** 10.3390/microorganisms10091719

**Published:** 2022-08-26

**Authors:** Yohei Sakai, Hiroshi Ochi, Miyuki Tanaka

**Affiliations:** Food Ingredients & Technology Institute, R&D Division, Morinaga Milk Industry Co., Ltd., 5-1-83 Higashihara, Zama, Kanagawa 252-8583, Japan

**Keywords:** constipation, daily change, immediate effect, indigestible oligosaccharide, intestinal microbiota, prebiotic

## Abstract

Oral administration of a low dose of lactulose increases the abundance of genus *Bifidobacterium* in the large intestine; however, the details of the daily variation in *Bifidobacterium* have not been researched. To observe how the intestinal microbiota, including *Bifidobacterium*, change, especially immediately after the initiation of ingestion, we conducted a randomised, placebo-controlled, double-blind crossover study of ingestion of 4 g lactulose/day for 2 weeks in 36 healthy Japanese (including males and females). The primary outcome was the percentage of *Bifidobacterium* in the faecal bacteria. In the lactulose-treatment group, the percentage of *Bifidobacterium* was already significantly higher 2 days after starting lactulose ingestion than in the placebo group (20.5 ± 1.2% vs. 17.1 ± 1.2%, *p* = 0.021). Significant differences were maintained, gradually widening, until the end of the 2-week intervention period. There were significant increases in the percentage and the number of *Bifidobacterium* with ingestion of 4 g lactulose/day for 2 weeks, but no significant changes in the beta diversity of the intestinal microbiota between lactulose and placebo ingestion. The percentage of *Bifidobacterium* in the faecal bacteria returned to its original level within a week of the end of intervention with lactulose.

## 1. Introduction

Many people in Japan suffer from constipation; according to the large-scale Comprehensive Survey of Living Conditions conducted in 2019, 3.48% of the general population answered that constipation was their most worrisome health concern [1]. In Japan, daily exercise is recommended to deal with constipation, and foods containing indigestible oligosaccharides are widely used, together with medicines such as magnesium oxide, as treatments. Lactulose is one such indigestible oligosaccharide. Ingestion of an appropriate amount of lactulose increases the amount of genus *Bifidobacterium* in the large intestine and improves the intestinal environment; a higher dose, acting as an osmotic laxative, improves defaecation [2]. In Japan, 4 g lactulose/day is used in the Food for Specified Health Uses and Food with Function Claims systems because it improves the intestinal environment of healthy people, even though the therapeutic dose for treatment of constipation is from around 15 g/day. Lactulose-containing products have a high sensory effect (i.e., the consumer is able to experience intestinal movement and enhanced defaecation), and the number of intestinal *Bifidobacterium* is believed to increase immediately after lactulose ingestion. Terada et al. reported that *Bifidobacterium* proliferated significantly on day 4 after ingestion of 3 g lactulose/day compared with before ingestion [3]. However, comparisons with placebo and information before day 4 were not reported.

Therefore, the present study observed daily changes in the gut microbiota, including in the abundance of *Bifidobacterium*, when 4 g lactulose/day was ingested. In addition, changes in the intestinal microbiota after the end of lactulose intake (i.e., during a washout period) were investigated.

## 2. Materials and Methods

### 2.1. Trial Design

**Figure 1:**
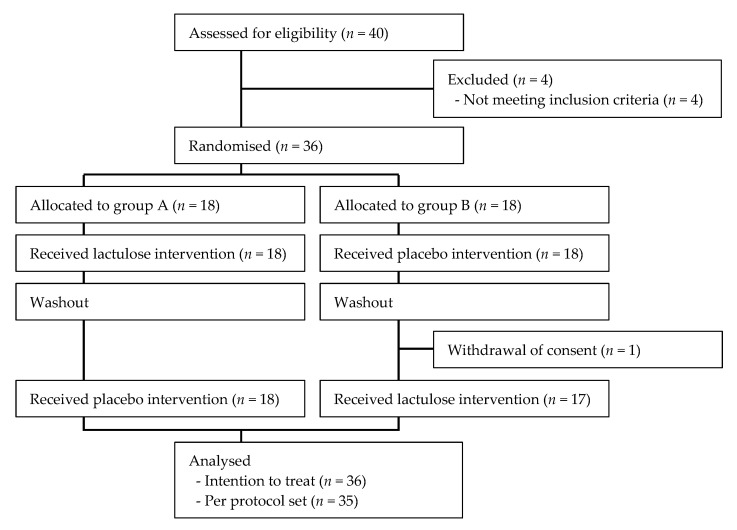
Flow chart of participant numbers throughout the trial.

This study was designed as a randomised, double-blind, placebo-controlled crossover trial consisting of a pre-observation period and then two ingestion periods separated by a washout period. The prebiotic effect of 4 g lactulose/day ingested for 2 weeks was evaluated (Figure 1). The study was conducted at the Megurodoori Gastrointestinal Surgery Internal Medicine Clinic, Tokyo, Japan, between May and October 2021, in accordance with the principles of the Declaration of Helsinki. The study protocol was approved by the Institutional Review Board of the Japan Conference of Clinical Research (Tokyo, Japan) (411). All participants provided written informed consent. The protocol for this study is registered with the University Hospital Medical Information Network Clinical Trials Registry (UMIN000044272).

### 2.2. Participants

Healthy men and women were recruited. The inclusion criteria were: (1) age 20–65 years; (2) defaecation frequency 3–7 days/week; and (3) having *Bifidobacterium* in the intestine. In addition, because the primary outcome of this clinical trial was to investigate the effect of ingestion of 4 g lactulose/day on the daily value of the percentage of *Bifidobacterium* in faeces, we recruited participants who were not constipated.

The exclusion criteria were: (1) a severe hepatic, renal, cardiac, gastrointestinal, cerebrovascular, endocrine, metabolic, or infectious disease; (2) a history of gastrointestinal resection; (3) gastrointestinal dysfunction, such as irritable bowel syndrome or inflammatory bowel disease; (4) the use of medicines or supplements that could influence defaecation frequency (e.g., antibiotics, probiotics, prebiotics, laxatives, antidiarrheal drugs, and fibre); (5) milk allergy and/or lactose intolerance; (6) participation in another study; and (7) individuals who were judged inappropriate for the study by the investigator or a physician (e.g., those who could not meet the deadline for submitting the survey diary or faecal samples or who could not be contacted during the test period).

### 2.3. Randomisation

Participants were randomly assigned to group A (lactulose first) or group B (placebo first) (Figure 1). The assignment manager was independent of the trial staff, and created the allocation order using the permutation block method (block size 4). The allocation ratio was 1:1. The trial staff assigned the test foods in ascending order of test food number, corresponding to participant number. Until completion of the trial, both the block size and the correspondence table showing the correspondence between the test food number and the assignment group were hidden from the authors, participants, and trial staff, including the intestinal microbiome analyst and the statistician.

### 2.4. Intervention

**Figure 2:**
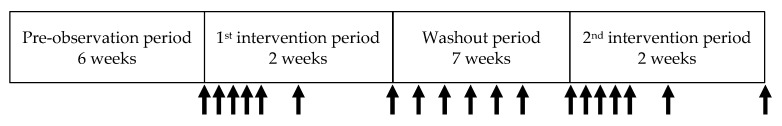
Trial design. Faecal sample collection is indicated with arrows.

Four-gram portions of lactulose crystal anhydrate powder (MLC-97, ≥97%; Morinaga Milk Industry Co., Ltd., Tokyo, Japan) were provided in aluminium sachets. Four-gram portions of glucose crystal anhydrate powder (Nihon Shokuhin Kako Co., Ltd., Tokyo, Japan) were used as the placebo. Glucose could not be discriminated from the lactulose. Participants in group A received lactulose during the first 2-week intervention period and the placebo during the second 2-week intervention period. Participants in group B received the placebo during the first intervention period and lactulose during the second intervention period. There was a 7-week washout period between the two intervention periods (Figure 2). The time of ingestion of test food was not specified, and participants consumed the test foods at their preferred time. The participants were instructed in advance to avoid the use of pharmaceuticals and supplements (e.g., antibiotics, laxatives, antidiarrheal drugs, probiotics, prebiotics, and fibre) that could affect defaecation during the study period.

### 2.5. Outcomes

The primary outcome was the percentage of *Bifidobacterium* in the faecal microbiome. Other outcomes were the *Bifidobacterium* cell number, defaecation frequency, the number of days on which defaecation occurred (defaecation days), faecal consistency, faecal volume, the degree of straining at defaecation (defaecation straining), and the time required for defaecation (defaecation time).

### 2.6. Faecal Sample Collection

Faecal samples were collected from participants just before first ingestion and 1 day, 2 days, 3 days, 4 days, 1 week, and 2 weeks after starting to take the test food (Figure 2). The faecal sample collection at 1 and 2 weeks was allowed ±2 days. Faecal samples were also collected once per week during the washout period.

Faecal samples were immediately placed in storage at <−18 °C in dedicated freezers until they arrived at the laboratory and stored at −30 °C thereafter. DNA was extracted from these samples by Techno-Suruga Laboratory Co., Ltd. (Shizuoka, Japan) and used to determine both the percentages of *Bifidobacterium* within the faecal microbiome and the cell numbers of *Bifidobacterium*.

### 2.7. DNA Extraction

The extraction of DNA was carried out with the following procedure, which is essentially the same as that reported by Takahashi et al. [4]. Frozen faecal samples were thawed on ice; 100 mg of each sample was suspended in 4 M guanidium thiocyanate, 100 mM Tris-HCl (pH 9.0), and 40 mM EDTA; and the samples were then beaten with zirconia beads using a Precellys Evolution instrument (Bertin Instruments, Montigny-le-Bretonneux, France). DNA was extracted from the bead-treated suspensions using a GENE PREP STAR PI-480 nucleic acid isolation system (Kurabo Industries Ltd., Osaka, Japan). DNA concentrations were estimated by spectrophotometry using an ND-1000 instrument (NanoDrop Technologies, Wilmington, DE, USA).

### 2.8. Percentage of Bifidobacterium in the Faecal Microbiome

The DNA extract was sequenced using Illumina MiSeq technology (Illumina, Inc., San Diego, CA, USA), essentially as described previously [5]. The V3-V4 region of the bacterial 16S rRNA gene was amplified by PCR in triplicate using the TaKaRa Ex Taq Hot Start Version (Takara Bio Inc., Shiga, Japan) and the primer sets Tru357F (5′-CGCTCTTCCGATCTCTGTACGGRAGGCAGCAG-3′) and Tru806R (5′-CGCTCTTCCGATCTGACGGACTACHVGGGTWTCTAAT-3′). A 1 μL sample of the combined PCR products was amplified with barcoded primers adapted for Illumina MiSeq sequencing: forward 5′-AATGATACGGCGACCACCGAGATCTACACXXXXXXXXACACTCTTTCCCTACACGACGCTCTTCCGATCTCTG-3′ and reverse 5′-CAAGCAGAAGACGGCATACGAGATXXXXXXXXGTGACTGGAGTTCAGACGTGTGCTCTTCCGATCTGAC-3′, where X represents a barcode base. The products were purified and quantified with a QIAquick PCR Purification Kit (Qiagen, Venlo, the Netherlands) and QuantiT PicoGreen dsDNA Assay Kit (Thermo Fisher Scientific, Waltham, MA, USA) according to the manufacturer’s protocols. Equal amounts of amplicons were pooled and purified with the GeneRead Size Selection Kit (Qiagen) according to the manufacturer’s protocol. The pooled libraries were sequenced with an Illumina MiSeq instrument and the MiSeq v3 Reagent Kit (Illumina, Inc.). After removing sequences consistent with data from the Genome Reference Consortium human build 38 (GRCh38) and PhiX reads from the raw Illumina paired-end reads, the sequences were analysed using the QIIME2 software package, version 2017.10 (https://qiime2.org/, accessed on 4 December 2017). Potential chimeric sequences were removed using DADA2 [6], then 30 and 90 bases of the 3′- regions of the forward and reverse reads were trimmed, respectively. Taxonomic classification was performed using a naive Bayes classifier trained on Greengenes 13.8, with a 99% threshold for operational taxonomic unit full-length sequences. UniFrac distances were calculated using QIIME2 software. The data collected at the end of the intervention period were statistically analysed.

### 2.9. Cell Numbers of Bifidobacterium

To determine the cell number of *Bifidobacterium*, the same DNA extract was used in a quantitative PCR method. Forward g-Bifid-F (5′-CTCCTGGAAACGGGTGG-3′) and reverse g-Bifid-R (5′-GGTGTTCTTCCCGATATCTACA-3′) primers were used as specific primers for the *Bifidobacterium* in the PCR [7]. A standard curve was prepared using dilutions of *B. longum* JCM1217^T^ (ATCC15707^T^) cells. The detection limit of the quantitative PCR was 6.74 (log colony-forming units (CFU)/g faeces).

### 2.10. Recording of Other Outcomes

Other outcomes were recorded in an electronic diary by the participants.

The mean weekly values for defaecation frequency were calculated using data from the entire intervention period for each participant. Days when participants defaecated at least once were counted as a ”defecation day”.

The Bristol Stool Form Scale (BSFS) was used for the evaluation of faecal consistency, using scores between 1 (hard) and 7 (watery) [8]. Mean faecal consistencies (BSFS value per defaecation) were calculated for the entire intervention period for each participant.

Faecal volume was measured using the faecal collection container ”Raku-Ryu Cup Wide” (Takahashi Keisei Corporation, Yamagata, Japan) as an index; a full container was assigned a value of 100. Mean weekly faecal volumes were calculated for the entire intervention period for each participant.

The record of defaecation straining was made using a visual analogue scale (VAS). Normally, VAS scores are assigned using a horizontal 10 cm long line, on which participants indicate their score by placing a cross on the line at the point that corresponds to their experience, with the left-hand side of the scale representing zero and the right-hand side representing the highest score they can imagine. In this study, the recording was undertaken on a smartphone, so the line was not exactly 10 cm long. Mean values of the VAS score were calculated for the entire intervention period for each participant.

Defaecation time was also recorded in the same electronic diary.

### 2.11. Statistical Methods

The number of participants required was calculated using G*Power 3.1 [9]. First, on the basis of the results of a preliminary trial of ingestion of 4 g lactulose/day in people who defaecated every day (unpublished), it was calculated to be 10 for a two-tailed significance level of 5%, a power of 80%, and effect size of 1.0 in the percentages of *Bifidobacterium* in the faecal microbiome between lactulose and placebo interventions. Next, if circumspectly assuming that the average defaecation frequency is 4 days per week, the probability that faeces can be collected on the same day after the start of ingestion in the first and second intervention periods is 16/49. Therefore, the required number of participants was 31, which was calculated by multiplying the calculated 10 participants by the reciprocal of 16/49. Assuming that the withdrawal and dropout rate would be about 10%, the required number of participants was rounded up to 35. Finally, as there were to be two groups, we decided on an even sample size of 36.

Statistical analysis was conducted on the basis of intention to treat (ITT). In addition, per protocol set (PPS) analysis was also performed as a sensitivity analysis, meaning that data for the participants who dropped out prematurely were excluded, and data obtained when participants took medications that could affect defaecation were excluded from the analysis.

The baselines and the diet survey were calculated as means ± standard deviation (SD) or frequency and analysed using an unpaired *t*-test, paired *t*-test, or Fisher’s exact test. The two-tailed significance level was set at 5%.

Comparison between lactulose and the placebo was made using a linear mixed model in which the test food group and timing were fixed effects, and participant identity was a random effect. The least mean square value and standard error (SE) for each group, the difference between groups, and the associated 95% confidence interval (CI) and *p*-value were calculated. The two-tailed significance level was set at 5%. The carryover effect was analysed using a model in which the interaction between the test food group and time was added to the fixed effect of the main analysis model. To avoid multiplicity, data after 2 weeks of intervention were set as endpoints, and then the test was performed retroactively from the closing procedure when evaluating the percentages and the cell numbers of *Bifidobacterium* in the faecal microbiota.

Comparisons before and after lactulose ingestion and before and after placebo ingestion were performed using a paired *t*-test. The mean and SD for each group and the *p*-value were calculated. The two-tailed significance level was set at 5%.

The relationship between the overall gut microbiome composition and lactulose ingestion was assessed using unweighted (qualitative) and weighted (quantitative) UniFrac distance matrices and permutational multivariate analysis of variance (PERMANOVA). The number of permutations was set to 10,000. Principal coordinate analysis (PCoA) plots were generated using the first two principal coordinates.

Differences in the frequency of adverse events between periods of lactulose and placebo ingestion were evaluated using McNemar’s test.

Statistical analyses of the data were mainly performed using JMP 14.0.0 software (SAS Institute Inc., Cary, NC, USA). Unweighted and weighted UniFrac distances obtained from the microbial data using the QIIME2 software package were used for PCoA. PERMANOVA was performed using the package ”vegan” in R software (version 3.6.0).

## 3. Results

### 3.1. Participants

Thirty-six healthy participants were enrolled in the study; 18 were assigned to group A and 18 to group B (Figure 1). There were 11 males and 25 females in the study. One female participant, belonging to group B, dropped out before starting the second intervention period. The mean age of the 36 participants was 41.7 ± 11.2 years (range, 21–61 years). Baseline data for the participants are shown in Table 1. There were no significant imbalances between groups A and B.

### 3.2. Compliance

The mean and SD of the ingestion rate for each participant was calculated as the compliance. The compliance (percentage ± SD) of group A was 99.3 ± 3.1% during the first intervention period and 99.3 ± 3.1% during the second intervention period. The compliance of group B was 98.8 ± 2.7% during the first intervention period and 99.2 ± 2.3% during the second intervention period. There was no significant imbalance between lactulose (99.2 ± 2.7%) and placebo ingestion (99.1 ± 2.9%; *p* = 0.99, Wilcoxon signed rank test).

### 3.3. Diet Survey

The diet survey showed no significant differences in the intakes of specific nutritional components before and at the end of the intervention (Table 2).

### 3.4. Percentage of Bifidobacterium in the Faecal Microbiome

**Figure A1:**
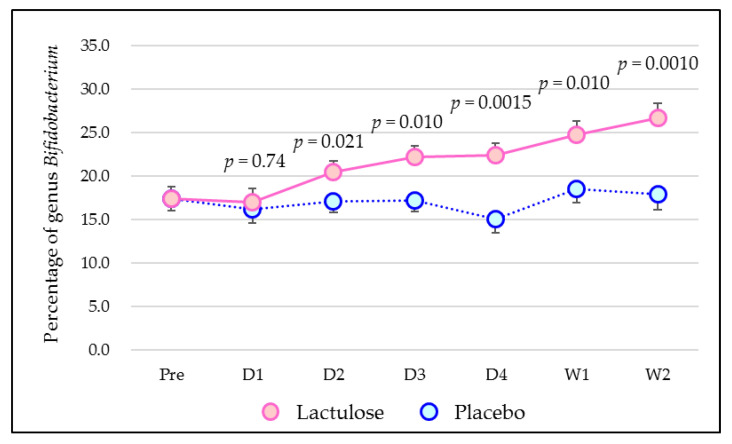
Percentages of genus *Bifidobacterium* in the faecal microbiome. Pre means just before first ingestion; D1 means 1 day after starting test food ingestion; D2 means 2 days after starting test food ingestion; D3 means 3 days after starting test food ingestion; D4 means 4 days after starting test food ingestion; W1 means 1 week after starting test food ingestion; and W2 means 2 weeks after starting test food ingestion. Values are expressed as the least square mean ± standard error (generalised linear mixed model).

**Figure A2:**
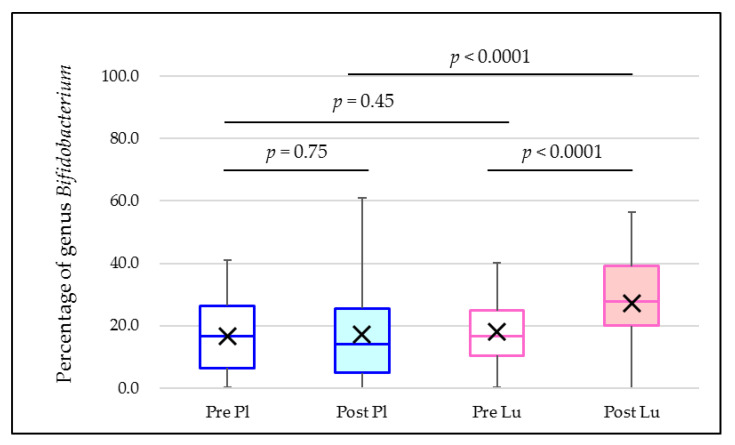
Comparison of the percentages of genus *Bifidobacterium* in faecal microbiota before intervention and after 2 weeks of intervention. Values are expressed as means (crosses) and quartiles. Pl, placebo; Lu, lactulose. *p*-values are for Pre Pl vs. Post Pl, Pre Lu vs. Post Lu, Pre Pl vs. Pre Lu, and Post Pl vs. Post Lu (paired *t*-test).

The transition in the percentage of *Bifidobacterium* in the faecal microbiome is shown in Table 3 and Appendix A Figure A1. In Table 3, the least square mean and SE were used to determine the percentage of *Bifidobacterium*. For the placebo ingestion, the value was 17.4 ± 1.4% just before ingestion, 17.1 ± 1.2% 2 days after starting ingestion, and 17.9 ± 1.7% 2 weeks after starting ingestion. For the lactulose ingestion, the value was 17.4 ± 1.4% before ingestion, 20.5 ± 1.2% 2 days after starting ingestion, and 26.7 ± 1.7% 2 weeks after starting ingestion. The differences between the percentages for the placebo and lactulose groups were 3.4 (95% CI 0.6–6.3, *p* = 0.021) 2 days after starting test food ingestion and 8.8 (95% CI 3.9–13.7, *p* = 0.0010) 2 weeks after starting test food ingestion. The interaction of the carryover effect of interventions across periods was not significant (*p* = 0.82). The transition of the percentage of *Bifidobacterium* in faecal microbiota during test food ingestion is shown in Appendix A Table A2, and the data for the comparison before intervention and after 2 weeks of intervention are shown in Appendix A Figure A2.

**Figure A3:**
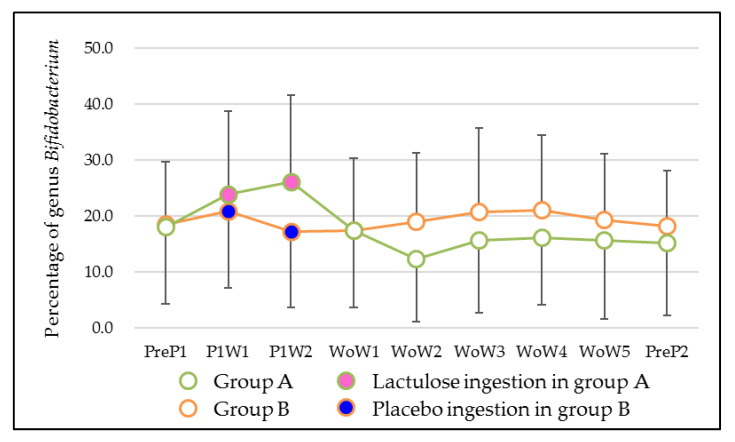
Transition in the percentages of genus *Bifidobacterium* in the faecal microbiome in a washout period. PreP1 means just before the first ingestion period; P1W1 means 1 week after starting the first period of test food ingestion; P1W2 means 2 weeks after starting the first period of test food ingestion; WoW1 means 1 week after finishing the first period of test food ingestion; WoW2 means 2 weeks after finishing the first period of test food ingestion; WoW3 means 3 weeks after finishing the first period of test food ingestion; WoW4 means 4 weeks after finishing the first period of test food ingestion; WoW5 means 5 weeks after finishing the first period of test food ingestion; and PreP2 means 7 weeks after finishing the first period of test food ingestion (and hence just before the second ingestion period). Group A ingested lactulose in the first intervention period, and group B ingested placebo in the first period. Values are expressed as the mean ± standard deviation.

The transition in the percentage of *Bifidobacterium* in the faecal microbiome in the washout period is shown in Table 4 and Appendix A Figure A3. In Table 4, the mean and SD are used. Group A ingested lactulose in the first intervention period. The mean for the percentage of *Bifidobacterium* in the faecal microbiome was 18.1 ± 13.7% just before the first ingestion period, 26.2 ± 15.4% 2 weeks after starting lactulose ingestion (and hence just before the washout period), and 17.4 ± 12.8% 1 week after finishing lactulose ingestion. One week after finishing lactulose ingestion, there was no significant differences compared to before the first ingestion period (*p* = 0.88), but a significant difference compared to the end of the ingestion period was observed (*p* = 0.036).

Group B ingested the placebo in the first intervention period. The mean value for the percentage of *Bifidobacterium* in the faecal microbiome was 18.5 ± 11.1% just before the first ingestion period, 20.8 ± 13.7% 1 week after starting placebo ingestion, 17.2 ± 13.5% 2 weeks after starting placebo ingestion (and hence just before the washout period), 17.3 ± 13.6% 1 week after finishing placebo ingestion, 19.0 ± 12.2% 2 weeks after finishing placebo ingestion, 20.7 ± 14.9% 3 weeks after finishing placebo ingestion, 21.0 ± 13.4% 4 weeks after finishing placebo ingestion, 19.2 ± 11.8% 5 weeks after finishing placebo ingestion, and 18.1 ± 10.0% 7 weeks after finishing placebo ingestion (and hence just before the second ingestion period).

Appendix AFigure A4 shows the results of the PCoA of the faecal microbiota after lactulose or placebo ingestion. The differences between the groups were not significant using unweighted UniFrac distances (*p* = 0.99) or weighted UniFrac distances (*p* = 0.99).

The succession of the percentages of the top 10 genera in the faecal microbiome before, during, and after the test food intervention is shown in Appendix A Table A1A–G (using the least square mean and SE). For bacteria other than the *Bifidobacterium*, no significant difference was observed between lactulose and placebo treatments, and the order of abundance of the top 10 genera was almost unchanged. The genus *Blautia* was predominant before the study food intervention, but lost this position to the *Bifidobacterium* in the lactulose-treatment group. However, no significant differences in the proportions of taxa other than *Bifidobacterium* were observed in the faecal microbiota from the lactulose or placebo intervention groups throughout the ingestion period.

### 3.5. Sensitivity Analysis

PPS analysis was performed as a sensitivity analysis. All or some of the data for five participants were excluded: (i) All data for one participant, who discontinued the study at her own request before starting the second intervention period, were excluded from the analysis. (ii) Data for the first week of the washout period were excluded for one participant who ingested the test food (for information, the test food that was ingested during the washout period was placebo). (iii) The pre-observation period data for one participant who was taking antibiotics early in the pre-observation period were excluded. (iv) Data for the sixth week of the washout period for one participant who took laxatives were excluded. (v) All data after the antibiotic intake for one participant who took antibiotics during the second intervention period were excluded. The results of the PPS analysis were fundamentally the same as those from the ITT analysis.

### 3.6. Other Outcomes

The transition in the cell numbers of *Bifidobacterium* is shown in Table 5 and Appendix A Figure A5; the least square mean and SE are used for the cell numbers of *Bifidobacterium*. In the placebo ingestion group, the cell number (log CFU/g faeces) was 10.27 ± 0.10 just before ingestion, 10.24 ± 0.11 4 days after starting ingestion, and 10.27 ± 0.10 2 weeks after starting ingestion. In the lactulose ingestion group the cell number (log CFU/g faeces) was 10.27 ± 0.10 just before ingestion, 10.63 ± 0.10 4 days after starting ingestion, and 10.59 ± 0.10 2 weeks after starting ingestion. The differences between the placebo and lactulose groups (in log CFU/g faeces) were 0.39 (95% CI 0.16–0.63, *p* = 0.0022) 4 days after starting test food ingestion, and 0.32 (95% CI 0.10–0.54, *p* = 0.0067) 2 weeks after starting test food ingestion. The transition in the cell numbers of *Bifidobacterium* during test food ingestion is shown in Appendix A Table A3, and the data for the comparison before intervention and after 2 weeks of the intervention are shown in Appendix A Figure A6.

No significant differences between lactulose and placebo intervention were observed in any indicators among the other outcomes except the cell numbers of *Bifidobacterium*.

### 3.7. Adverse Events

No side effects or serious adverse events were observed in any of the participants. The main secondary symptoms were gastrointestinal symptoms, but there were no significant differences in the incidence of these between the periods of lactulose and placebo ingestion (Table 6 and Table 7).

## 4. Discussion

We observed the daily changes in the abundance of *Bifidobacterium* in the faecal microbiota on oral administration of 4 g lactulose/day. The difference in the percentage of *Bifidobacterium* in the faecal bacteria between lactulose ingestion and placebo ingestion was not significant 1 day after starting ingestion, but it was significant at 2 days after, and the difference gradually increased until 2 weeks after the start of ingestion (Table 3). This result is consistent with the previous report by Terada et al. [3]. According to Kawai et al. [10], the gastrointestinal transit time of Japanese is 45.0 ± 26.3 h, which seems to be consistent with our results. We suggest that lactulose increased the abundance of *Bifidobacterium* at almost the same time it reached the cecum. The increase in the percentage of *Bifidobacterium* was then observed when the large intestine contents with increased *Bifidobacterium* were excreted from the body. However, it was not possible to determine whether the gradual increase in the mean difference between the lactulose and placebo groups was due to the time it took for the *Bifidobacterium* in each participant to increase or to an immediate increase in the count of *Bifidobacterium* in some participants versus a lack of increase in others. This is because none of the individual data showed a clear upward trend like the average in Appendix A Figure A1. However, it might be presumed that the percentage of *Bifidobacterium* gradually increased in each participant because most of the participants submitted faecal samples on the expected schedule.

When lactulose intake was stopped, the level of occupancy of *Bifidobacterium* returned to the original level within 1 week of the end of the intervention (Table 4A, Appendix A Figure A3). Therefore, it is desirable to continue to take lactulose to continue to benefit from the proliferative effect of *Bifidobacterium* of lactulose. In addition, lactulose continued to increase *Bifidobacterium* gradually over the 2 week ingestion period; however, it was not possible to determine whether or not the upper limit of the abundance of *Bifidobacterium* was reached. Therefore, it is not possible to conclude from the results of this trial alone how *Bifidobacterium* would increase if lactulose ingestion were continued for >2 weeks.

**Figure A4:**
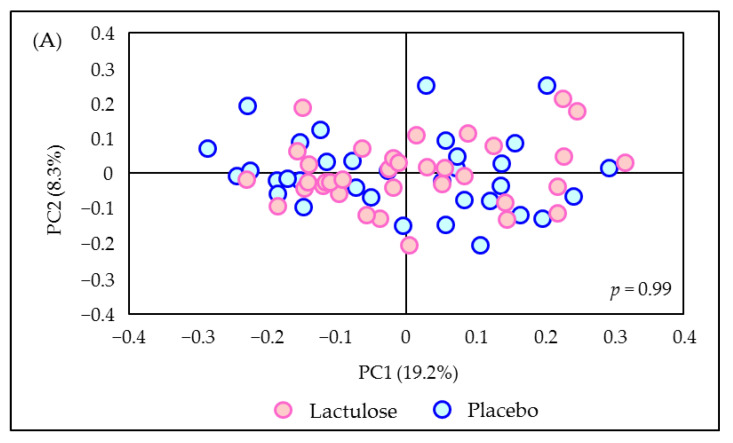

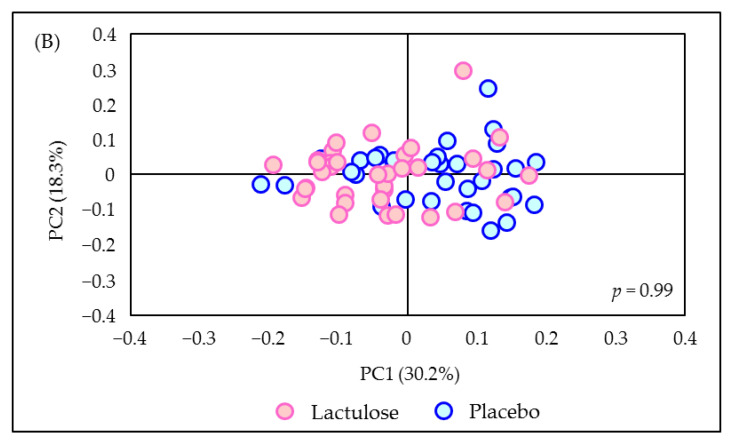
Principal coordinate analysis score plots generated using the first two principal coordinates (PC1 and PC2) based on data on the abundance of intestinal microbiota. Data were analysed using permutational multivariate analysis of variance and were based on (**A**) unweighted UniFrac distances or (**B**) weighted UniFrac distances. The differences between groups were not significant using the unweighted UniFrac distances (*p* = 0.99) or weighted UniFrac distances (*p* = 0.99).

**Figure A5:**
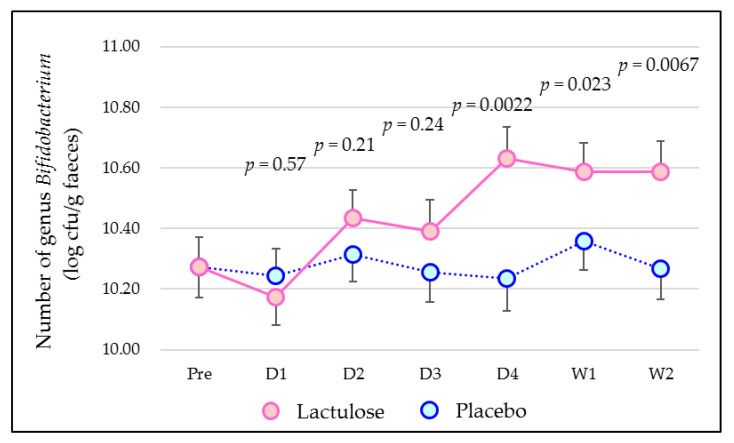
Cell numbers of genus *Bifidobacterium* in faeces. Pre means just before ingestion; D1 means 1 day after starting test food ingestion; D2 means 2 days after starting test food ingestion; D3 means 3 days after starting test food ingestion; D4 means 4 days after starting test food ingestion; W1 means 1 week after starting test food ingestion; and W2 means 2 weeks after starting test food ingestion. Values are expressed as the least square mean ± standard error (generalised linear mixed model). CFU, colony-forming units.

**Figure A6:**
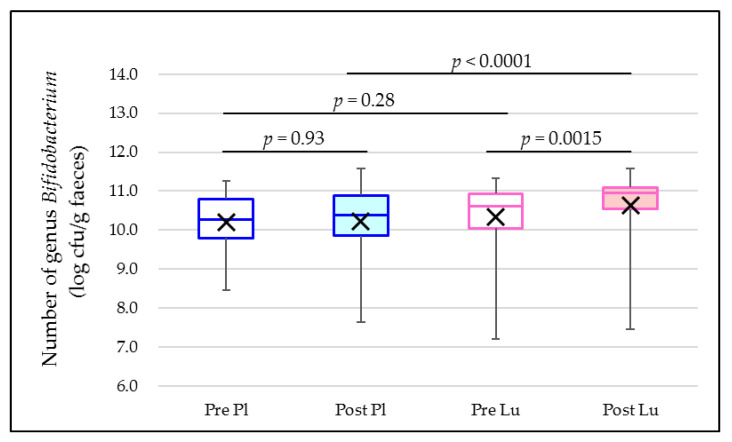
Comparison of the cell numbers of genus *Bifidobacterium* in faeces before intervention and after 2 weeks of intervention. Values are expressed as means (crosses) and quartiles. Pl, placebo; Lu, lactulose. *p*-values are for Pre Pl vs. Post Pl, Pre Lu vs. Post Lu, Pre Pl vs. Pre Lu, and Post Pl vs. Post Lu (paired *t*-test). CFU, colony-forming units.

**Figure A7:**
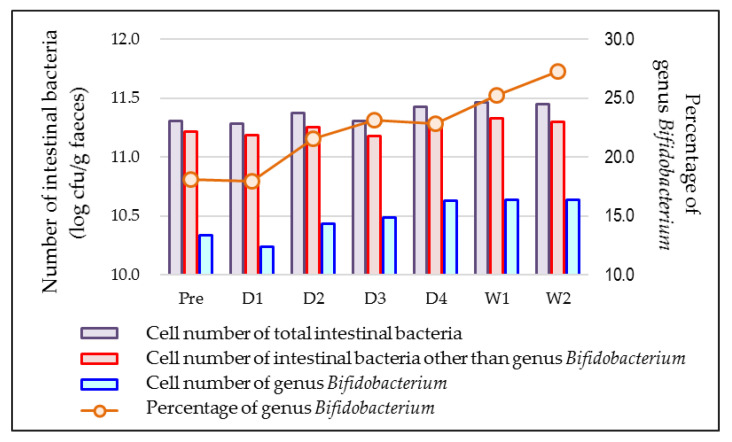
Cell numbers of intestinal bacteria and percentages of genus *Bifidobacterium* during lactulose ingestion. Pre means just before lactulose ingestion; D1 means 1 day after starting lactulose ingestion; D2 means 2 days after starting lactulose ingestion; D3 means 3 days after starting lactulose ingestion; D4 means 4 days after starting lactulose ingestion; W1 means 1 week after starting lactulose ingestion; and W2 means 2 weeks after starting lactulose ingestion. Values are expressed as the mean. CFU, colony-forming units.

On ingestion of lactulose, the cell numbers of *Bifidobacterium* increased daily in the same manner as the percentage of *Bifidobacterium*, and a significant difference between lactulose ingestion and placebo ingestion was observed after the fourth day (Appendix A Figure A5). No significant changes in the abundance of high-occupancy intestinal bacteria other than *Bifidobacterium* were observed on lactulose ingestion (Table A1A–G). The significant increase of *Bifidobacterium* due to lactulose ingestion did not affect the beta diversity of the intestinal microbiota (Appendix A Figure A4). As there was a time lag between the percentages of *Bifidobacterium* and the cell numbers of *Bifidobacterium*, the total number of intestinal bacteria during lactulose ingestion was tentatively calculated from our data on the cell numbers and the percentages of *Bifidobacterium* (Appendix A Figure A7). As a result, we suggest that this time lag was caused by an increase in the cell numbers of intestinal bacteria other than *Bifidobacterium* resulting from the lactulose ingestion.

These results are partly the same as, and partly different from, those in our previous study of ingestion of 2 g lactulose/day (the present study used 4 g lactulose/day) [11]. The effects of lactulose on the intestinal microbiota, including proliferation of *Bifidobacterium*, were similar. However, in contrast to the previous study, there were no significant differences in the present study between the lactulose and placebo ingestion groups in the other outcomes (such as those relating to defaecation). We suggest that there are three main reasons for the difference in the defaecation frequency between the previous study and the present study. First, there were not many participants in this study who had margins for increased defaecation frequency. In the previous study, only participants with a tendency to constipation (i.e., those who defaecated 2 to 4 times per week) were recruited, but the relevant inclusion criterion for the present study was defaecation 3 to 7 days per week. Thus, because many of the present participants already defaecated almost every day, there was little margin to increase their defaecation frequency. Second, in the previous study, the ingestion period began after participants had faecal samples taken, and further faecal samples were then taken after the end of 2 weeks of ingestion. The effect of this sampling methodology on the defaecation habit is estimated to be small. However, in the current trial, the participants were asked to collect faecal samples daily for the first 5 days. It might be that increased defaecation frequency occurred because of the conscious collection of faecal samples for the study, differing from normal defaecation habits, which eliminated the difference in defaecation frequency between the placebo ingestion and lactulose ingestion groups. In fact, a significant increase in defaecation days was observed between the pre-observation period and the first investigation period, especially in group B (placebo intervention first; *p* = 0.014, paired *t*-test). Third, the number of participants in this trial was less than the number required to assess defaecation frequency. This is because the current trial was focused on observing the transition in the percentages of *Bifidobacterium* in the intestinal microbiota when ingesting 4 g lactulose/day, whereas the previous study focused on observing differences in defaecation frequency between lactulose and placebo ingestion. On the basis of the results of our previous trial of ingestion of 2 g lactulose/day in people with a defaecation frequency of 2–4 times/week [11], the number of participants required was calculated to be 58 using G*Power 3.1 for a two-tailed significance level of 5%, a power of 80%, and an effect size of 0.38 for the defaecation frequency between lactulose and placebo interventions. In addition to these considerations, the mean age and male-to-female ratio of the participants in the present work were different from those in the previous study, which might have been influencing factors for the outcomes. The reason the faecal consistency did not change significantly between lactulose and placebo ingestion in this trial was that the faecal consistency of the participants during the pre-observation period was normal (BSFS score 4.0 ± 0.8.) Another interpretation is that that 4 g lactulose/day does not cause the faeces to soften beyond normal. If the faeces is not hard (i.e., if the participant is not suffering from constipation), the faecal volume and the defaecation straining would be unlikely to change significantly on treatment. If the defaecation straining does not change, the defaecation time is unlikely to differ significantly. Therefore, we think the present results from ingestion of 4 g lactulose/day differed from the previous study of ingestion of 2 g lactulose/day [11] because, here, we focused on observing daily changes in occupancy of *Bifidobacterium*, and the design of this trial was not suitable as a test system for evaluating the effect of lactulose on defaecation frequency or properties.

Increase in *Bifidobacterium*, which are representative of beneficial intestinal bacteria, in the large intestine is a phenotype suggesting improvement of the intestinal environment. Although it has been known for 65 years that lactulose increases the number of *Bifidobacterium* [12], the present study shows that the effect is rapid, even with a small ingestion of 4 g lactulose/day. In other words, as soon as lactulose reached the entrance of the large intestine, it transformed the area into a layer of intestinal contents dominated by *Bifidobacterium*. It is often said that lactulose ingestion provides a “sensory effect” within a short period (a few hours after ingestion), which is generally thought to be caused by the physical irritation to the intestinal tract of hydrogen and carbon dioxide gases generated by the metabolism of lactulose by intestinal bacteria living at the entrance of the large intestine, including the cecum. In addition, irritation of the intestinal tract due to rapid changes in the concentrations of metabolites that bring about low pH, such as short-chain fatty acids, following the rapid growth of *Bifidobacterium* might also be one of the mechanisms of the sensory effect caused by lactulose. 

Lactulose is widely used worldwide as an osmotic laxative, but it is not intended to promote the growth of *Bifidobacterium*. When lactulose is ingested as a prebiotic for the growth of *Bifidobacterium*, it is important to avoid the risk of diarrhoea; that is, to increase *Bifidobacterium* using small amounts of lactulose. We expect that the prebiotic effect of lactulose at doses as low as 4 g/day can contribute to the health of many people.

## 5. Conclusions

To observe how the intestinal microbiota, including *Bifidobacterium*, changes, especially immediately after the initiation of ingestion, we conducted a randomised, placebo-controlled, double-blind crossover study of ingestion of 4 g lactulose/day for 2 weeks in 36 healthy Japanese without constipation. The percentage of *Bifidobacterium* in the faecal microbiota was already significantly higher with lactulose treatment than with placebo treatment 2 days after starting lactulose ingestion, and significant differences were maintained, gradually widening until the end of the 2 week intervention period. The abundance of *Bifidobacterium* returned to the original level within 1 week of the end of intervention.

## Figures and Tables

**Table 1:** Anthropometric data at the start of the trial ^1^.

Parameter	Whole Cohort	Group A (Lactulose First)	Group B (Placebo First)	*p*-Value
	*n* = 36	*n* = 18	*n* = 18	
Sex (female/male)	25/11	11/7	14/4	0.47 ^3^
Age (years)	41.7 ± 11.2	42.1 ± 12.1	41.3 ± 10.5	0.84 ^4^
Body mass (kg)	57.7 ± 11.0	59.0 ± 13.3	56.5 ± 8.1	0.51 ^4^
Height (m)	1.61 ± 0.07	1.61 ± 0.08	1.61 ± 0.07	0.94 ^4^
BMI ^2^ (kg/m^2^)	22.2 ± 3.1	22.6 ± 3.7	21.8 ± 2.3	0.45 ^4^
Defaecation frequency (days/week)	4.9 ± 1.4	5.2 ± 1.3	4.6 ± 1.3	0.20 ^4^
Past medical history ^5^ (Y/N)	0:36	0:18	0:18	-
Morbidities (Y/N)	0:36	0:18	0:18	-
Smoking (>1 cigarette per week) (Y/N)	3:33	2:16	1:17	1.00 ^3^
Drinking any alcohol (>2 times per week) (Y/N)	8:28	3:15	5:13	0.69 ^3^

^1^ Values are expressed as the mean ± standard deviation. ^2^ BMI, body mass index. ^3^ Fisher’s exact test. ^4^ Unpaired *t*-test. ^5^ Previous problems with the participants’ health.

**Table 2:** Diet survey ^1^.

Parameter	*n*	Before the Intervention ^3^	*n*	At the End of the Intervention ^3^	*p*-Value ^2^
Energy (kcal/day)	36	1390 ± 514	35	1399 ± 572	0.97
Mass (g/day)	36	1679 ± 708	35	1673 ± 737	0.88
Water (g/day)	36	1379 ± 612	35	1370 ± 632	0.83
Protein (g/day)	36	54.8 ± 19.0	35	55.5 ± 21.0	0.85
Fat (g/day)	36	44.4 ± 17.1	35	44.1 ± 19.2	0.79
Carbohydrate (g/day)	36	181.2 ± 82.2	35	183.7 ± 84.3	0.89
Soluble dietary fibre (g/day)	36	2.2 ± 1.4	35	2.4 ± 1.4	0.58
Insoluble dietary fibre (g/day)	36	6.3 ± 3.4	35	6.6 ± 3.6	0.47
Total dietary fibre (g/day)	36	8.8 ± 4.9	35	9.2 ± 5.2	0.53

^1^ Values are expressed as the mean ± standard deviation (paired *t*-test). ^2^ The paired *t*-test was performed, except for the data for one participant who dropped out. ^3^ A diet survey was conducted before the first intervention period and at the end of the second intervention period using a brief self-administered diet history questionnaire.

**Table 3:** Transition in the percentage of genus *Bifidobacterium* in the faecal microbiome ^1^.

Time of Sampling	*n*	Placebo	Lactulose	Difference ^2^ (95% CI ^3^)	*p*-Value
Just before the ingestion period	36	17.4 ± 1.4	17.4 ± 1.4	-	-
One day after starting test food ingestion	36	16.2 ± 1.6	17.0 ± 1.6	0.8 (−3.9 to 5.5)	0.74
Two days after starting test food ingestion	36	17.1 ± 1.2	20.5 ± 1.2	3.4 (0.6 to 6.3)	0.021
Three days after starting test food ingestion	36	17.2 ± 1.2	22.2 ± 1.3	5.0 (1.3 to 8.7)	0.010
Four days after starting test food ingestion	36	15.0 ± 1.5	22.4 ± 1.5	7.4 (3.1 to 11.6)	0.0015
One week after starting test food ingestion	36	18.5 ± 1.5	24.8 ± 1.5	6.3 (1.6 to 11.0)	0.010
Two weeks after starting test food ingestion	36	17.9 ± 1.7	26.7 ± 1.7	8.8 (3.9 to 13.7)	0.0010

^1^ Values are expressed as the least square mean ± standard error (generalised linear mixed model). ^2^ Lactulose minus placebo. ^3^ CI, confidence interval.

**Table 4:** (**A**) Transition in the percentages of genus *Bifidobacterium* in the faecal microbiomes of group A consuming lactulose ^1,2^. (**B**) Transition in the percentages of genus *Bifidobacterium* in the faecal microbiomes of group B consuming placebo ^1,2^.

Time of Sampling (A)	*n* ^3^	Percentage of Genus *Bifidobacterium*	*p*-Value vs. before the First Ingestion Period	*p*-Value vs. Just before the Washout Period
Just before the first ingestion period	18	18.1 ± 13.7	-	-
One week after starting lactulose ingestion	18	23.8 ± 15.0	0.0094	-
Just before the washout period ^4^	18	26.2 ± 15.4	0.0040	-
One week after the end of lactulose ingestion	17	17.4 ± 12.8	0.88	0.036
Two weeks after the end of lactulose ingestion	17	12.2 ± 11.1	0.12	0.0002
Three weeks after the end of lactulose ingestion	18	15.6 ± 13.0	0.41	0.0032
Four weeks after the end of lactulose ingestion	18	16.1 ± 12.0	0.41	0.0018
Five weeks after the end of lactulose ingestion	18	15.5 ± 13.9	0.30	0.0016
Seven weeks after the end of lactulose ingestion ^5^	18	15.2 ± 13.0	0.30	0.0012
**Time of Sampling (B)**	***n*** **^3^**	**Percentage of Genus *Bifidobacterium***	***p*** **-Value vs. before the First Ingestion Period**	***p*** **-Value vs. Just before the Washout Period**
Just before the first ingestion period	17	18.5 ± 11.1	-	-
One week after starting placebo ingestion	17	20.8 ± 13.7	0.11	-
Just before the washout period ^4^	17	17.2 ± 13.5	0.86	-
One week after the end of placebo ingestion	17	17.3 ± 13.6	0.94	0.96
Two weeks after the end of placebo ingestion	17	19.0 ± 12.2	0.40	0.29
Three weeks after the end of placebo ingestion	17	20.7 ± 14.9	0.30	0.073
Four weeks after the end of placebo ingestion	17	21.0 ± 13.4	0.17	0.027
Five weeks after the end of placebo ingestion	17	19.2 ± 11.8	0.48	0.23
Seven weeks after the end of placebo ingestion ^5^	17	18.1 ± 10.0	0.80	0.62

For (**A**) ^1^ Group A ingested lactulose in the first intervention period. ^2^ Values are expressed as the mean ± standard deviation (paired *t*-test). ^3^ The paired *t*-test was performed, except for missing data (no faecal samples were submitted for one person at 1 and 2 weeks after the end of lactulose ingestion). ^4^ Two weeks after starting lactulose ingestion. ^5^ Just before the second ingestion period. For (**B**) ^1^ Group B ingested placebo in the first intervention period. ^2^ Values are expressed as the mean ± standard deviation (paired *t*-test). ^3^ One participant in Group B who dropped out before the start of the second intervention period did not submit a faecal sample, so analysis was performed for 17 participants. ^4^ Two weeks after starting placebo ingestion. ^5^ Just before the second ingestion period.

**Table 5:** Transition in the cell numbers of genus *Bifidobacterium* in the faeces ^1^.

Time of Sampling	*n*	Placebo	Lactulose	Difference ^2^ (95% CI ^3^)	*p*-Value
Just before the ingestion period	36	10.27 ± 0.10	10.27 ± 0.10	-	-
One day after starting test food ingestion	36	10.24 ± 0.09	10.17 ± 0.09	−0.07 (−0.33 to 0.18)	0.57
Two days after starting test food ingestion	36	10.32 ± 0.09	10.43 ± 0.09	0.12 (−0.07 to 0.31)	0.21
Three days after starting test food ingestion	36	10.26 ± 0.10	10.39 ± 0.10	0.13 (−0.09 to 0.36)	0.24
Four days after starting test food ingestion	36	10.24 ± 0.11	10.63 ± 0.10	0.39 (0.16 to 0.63)	0.0022
One week after starting test food ingestion	36	10.36 ± 0.10	10.59 ± 0.09	0.23 (0.03 to 0.43)	0.023
Two weeks after starting test food ingestion	36	10.27 ± 0.10	10.59 ± 0.10	0.32 (0.10 to 0.54)	0.0067

^1^ Values are expressed as the least square mean ± standard error (generalised linear mixed model). ^2^ Lactulose minus placebo. ^3^ CI, confidence interval.

**Table 6:** Adverse events: principal secondary abdominal symptoms ^1^.

Abdominal Symptom	Lactulose	Placebo
Stomach ache	0	0
Heartburn	0	0
Abdominal pain	2	1
Diarrhoea	3	1
Constipation	0	0
Trapped wind	0	0
Abdominal fullness	0	5
Flatus	3	2
Total	8	9

^1^ The number of significant secondary abdominal symptoms during each intervention.

**Table 7:** Adverse effects in the form of gastrointestinal symptoms ^1^.

		Lactulose			*p*-Value
		Number Not Reporting	Number Reporting	Total	
Placebo	Number not reporting	27	2	29	0.41
	Number reporting	4	3	7	
	Total	31	5	36	

^1^ McNemar’s test was performed for the presence or absence of adverse events between treatment groups.

## Data Availability

Not applicable.

## Appendix A

**Table A1:** (**A**). Percentages in the faecal microbiome before the test food intervention ^1^. (**B**). Percentages of the faecal microbiome 1 day after the start of test food ingestion ^1^. (**C**). Percentages of the faecal microbiome 2 days after the start of test food ingestion ^1^. (**D**). Percentages of the faecal microbiome 3 days after the start of test food ingestion ^1^. (**E**). Percentages of the faecal microbiome 4 days after the start of test food ingestion ^1^. (**F**). Percentages of the faecal microbiome 1 week after the start of test food ingestion ^1^. (**G**). Percentages of the faecal microbiome 2 weeks after the start of test food ingestion ^1^.

Genus (A)	*n*	Placebo	Lactulose	Difference ^2^ (95% CI ^3^)	*p*-Value
*Blautia*	36	23.3 ± 1.2	23.3 ± 1.2	-	-
*Bifidobacterium*	36	17.4 ± 1.4	17.4 ± 1.4	-	-
*Coprococcus*	36	7.6 ± 0.9	7.6 ± 0.9	-	-
Lachnospiraceae genus (*Ruminococcus*) ^4^	36	6.6 ± 0.8	6.6 ± 0.8	-	-
*Streptococcus*	36	6.5 ± 1.4	6.5 ± 1.4	-	-
Lachnospiraceae gen. ^4^	36	4.2 ± 0.4	4.2 ± 0.4	-	-
*Faecalibacterium*	36	4.2 ± 0.6	4.2 ± 0.6	-	-
*Ruminococcus*	36	4.1 ± 0.6	4.1 ± 0.6	-	-
*Collinsella*	36	2.6 ± 0.3	2.6 ± 0.3	-	-
*Gemmiger*	36	2.0 ± 0.3	2.0 ± 0.3	-	-
**Genus (B)**	***n***	**Placebo**	**Lactulose**	**Difference ^2^ (95% CI ^3^)**	***p*** **-Value**
*Blautia*	36	22.3 ± 1.2	23.5 ± 1.3	1.2 (−2.3 to 4.9)	0.47
*Bifidobacterium*	36	16.2 ± 1.6	17.0 ± 1.6	0.8 (−3.9 to 5.5)	0.74
*Coprococcus*	36	7.9 ± 0.5	7.9 ± 0.5	−0.0 (−1.4 to 1.4)	0.99
Lachnospiraceae genus (*Ruminococcus*) ^4^	36	6.1 ± 0.7	5.2 ± 0.7	−0.9 (−3.6 to 1.8)	0.51
*Streptococcus*	36	5.3 ± 0.9	6.3 ± 0.9	1.0 (−1.9 to 4.0)	0.49
Lachnospiraceae gen. ^4^	36	4.4 ± 0.5	4.1 ± 0.5	−0.3 (−1.6 to 1.1)	0.67
*Faecalibacterium*	36	3.4 ± 0.5	3.3 ± 0.6	−0.1 (−1.8 to 1.5)	0.83
*Ruminococcus*	36	5.1 ± 0.7	4.9 ± 0.7	−0.2 (−1.9 to 1.6)	0.84
*Collinsella*	36	2.7 ± 0.3	2.6 ± 0.3	−0.1 (−1.0 to 0.9)	0.88
*Gemmiger*	36	3.0 ± 0.6	3.3 ± 0.6	0.3 (−0.9 to 1.5)	0.61
**Genus (C)**	***n***	**Placebo**	**Lactulose**	**Difference ^2^ (95% CI ^3^)**	***p*** **-Value**
*Blautia*	36	21.5 ± 1.2	19.2 ± 1.2	−2.3 (−4.7 to 0.1)	0.062
*Bifidobacterium*	36	17.1 ± 1.2	20.5 ± 1.2	3.4 (0.6 to 6.3)	0.021
*Coprococcus*	36	9.0 ± 0.6	8.2 ± 0.6	−0.8 (−2.3 to 0.6)	0.23
Lachnospiraceae genus (*Ruminococcus*) ^4^	36	6.4 ± 0.5	5.9 ± 0.5	−0.5 (−1.8 to 0.8)	0.45
*Streptococcus*	36	5.3 ± 1.0	5.4 ± 1.0	0.1 (−2.9 to 3.2)	0.93
Lachnospiraceae gen. ^4^	36	4.7 ± 0.6	4.5 ± 0.7	−0.2 (−2.0 to 1.6)	0.79
*Faecalibacterium*	36	4.5 ± 0.6	4.4 ± 0.6	−0.1 (−2.0 to 1.9)	0.96
*Ruminococcus*	36	4.4 ± 0.7	3.7 ± 0.7	−0.7 (−3.0 to 1.6)	0.53
*Collinsella*	36	2.4 ± 0.4	3.0 ± 0.4	0.6 (−0.5 to 1.7)	0.30
*Gemmiger*	36	1.9 ± 0.6	2.5 ± 0.6	0.6 (−1.7 to 2.9)	0.59
**Genus (D)**	***n***	**Placebo**	**Lactulose**	**Difference ^2^ (95% CI ^3^)**	***p*** **-Value**
*Blautia*	36	23.2 ± 1.5	19.8 ± 1.6	−3.4 (−7.1 to 0.3)	0.067
*Bifidobacterium*	36	17.2 ± 1.2	22.2 ± 1.3	5.0 (1.3 to 8.7)	0.010
*Coprococcus*	36	7.6 ± 0.6	6.5 ± 0.6	−1.1 (−2.3 to 0.3)	0.11
Lachnospiraceae genus (*Ruminococcus*) ^4^	36	5.5 ± 1.0	6.3 ± 1.0	0.8 (−1.5 to 3.1)	0.49
*Streptococcus*	36	4.7 ± 0.7	3.6 ± 0.7	−1.1 (−3.4 to 1.1)	0.30
Lachnospiraceae gen. ^4^	36	4.2 ± 0.5	3.8 ± 0.5	−0.4 (−2.0 to 1.3)	0.68
*Faecalibacterium*	36	5.1 ± 0.6	3.6 ± 0.6	−1.5 (−3.8 to 0.8)	0.19
*Ruminococcus*	36	5.1 ± 0.6	3.9 ± 0.7	−1.2 (−2.9 to 0.4)	0.13
*Collinsella*	36	2.5 ± 0.5	3.3 ± 0.5	0.8 (−0.8 to 2.4)	0.30
*Gemmiger*	36	2.5 ± 0.3	1.9 ± 0.3	−0.6 (−1.5 to 0.2)	0.12
**Genus (E)**	***n***	**Placebo**	**Lactulose**	**Difference ^2^ (95% CI ^3^)**	***p*** **-Value**
*Blautia*	36	21.6 ± 1.5	22.5 ± 1.5	0.9 (−2.0 to 3.7)	0.53
*Bifidobacterium*	36	15.0 ± 1.5	22.4 ± 1.5	7.4 (3.1 to 11.6)	0.0015
*Coprococcus*	36	8.4 ± 0.5	7.2 ± 0.5	−1.2 (−2.7 to 0.3)	0.10
Lachnospiraceae genus (*Ruminococcus*) ^4^	36	5.9 ± 0.7	4.6 ± 0.7	−1.3 (−2.9 to 0.3)	0.10
*Streptococcus*	36	5.9 ± 1.0	2.8 ± 1.0	−3.1 (−6.7 to 0.5)	0.085
Lachnospiraceae gen. ^4^	36	5.9 ± 0.7	5.2 ± 0.6	−0.7 (−2.2 to 0.9)	0.41
*Faecalibacterium*	36	3.8 ± 0.6	4.6 ± 0.6	0.8 (−0.8 to 2.6)	0.29
*Ruminococcus*	36	4.4 ± 0.6	3.5 ± 0.6	−0.9 (−2.6 to 0.7)	0.25
*Collinsella*	36	2.4 ± 0.8	3.5 ± 0.7	1.1 (−0.8 to 3.1)	0.24
*Gemmiger*	36	2.2 ± 0.7	2.7 ± 0.7	0.5 (−0.4 to 1.6)	0.24
**Genus (F)**	***n***	**Placebo**	**Lactulose**	**Difference ^2^ (95% CI ^3^)**	***p*** **-Value**
*Blautia*	36	23.9 ± 1.6	20.4 ± 1.5	−3.5 (−7.6 to 0.6)	0.092
*Bifidobacterium*	36	18.5 ± 1.5	24.8 ± 1.5	6.3 (1.6 to 11.0)	0.010
*Coprococcus*	36	8.1 ± 0.7	6.6 ± 0.6	−1.5 (−2.8 to −0.1)	0.031
Lachnospiraceae genus (*Ruminococcus*) ^4^	36	5.4 ± 0.5	4.5 ± 0.5	−0.9 (−2.5 to 0.6)	0.21
*Streptococcus*	36	4.7 ± 0.9	3.5 ± 0.9	−1.2 (−4.2 to 1.8)	0.42
Lachnospiraceae gen. ^4^	36	4.0 ± 0.5	4.5 ± 0.4	0.5 (−1.0 to 2.0)	0.49
*Faecalibacterium*	36	4.1 ± 0.5	4.4 ± 0.5	0.3 (−1.3 to 2.0)	0.66
*Ruminococcus*	36	3.7 ± 0.6	4.0 ± 0.6	0.3 (−2.0 to 2.5)	0.80
*Collinsella*	36	2.8 ± 0.9	3.7 ± 0.8	0.9 (−1.5 to 3.3)	0.46
*Gemmiger*	36	3.2 ± 0.7	2.7 ± 0.7	−0.5 (−2.0 to 1.0)	0.50
**Genus (G)**	***n***	**Placebo**	**Lactulose**	**Difference ^2^ (95% CI ^3^)**	***p*** **-Value**
*Blautia*	36	23.8 ± 1.8	21.5 ± 1.8	−2.3 (−6.7 to 2.1)	0.30
*Bifidobacterium*	36	17.9 ± 1.7	26.7 ± 1.7	8.8 (3.9 to 13.7)	0.0010
*Coprococcus*	36	7.7 ± 0.7	6.2 ± 0.7	−1.5 (−3.8 to 0.7)	0.18
Lachnospiraceae genus (*Ruminococcus*) ^4^	36	5.5 ± 0.6	4.6 ± 0.6	−0.9 (−2.6 to 0.8)	0.29
*Streptococcus*	36	4.4 ± 1.0	4.1 ± 1.0	−0.3 (−2.7 to 2.1)	0.79
Lachnospiraceae gen. ^4^	36	5.4 ± 0.6	4.7 ± 0.6	−0.7 (−2.3 to 0.8)	0.32
*Faecalibacterium*	36	4.7 ± 0.7	4.7 ± 0.7	−0.0 (−0.0 to 0.0)	1.00
*Ruminococcus*	36	4.2 ± 0.6	3.6 ± 0.6	−0.6 (−1.6 to 0.5)	0.31
*Collinsella*	36	2.5 ± 0.4	3.0 ± 0.4	0.5 (−0.7 to 1.7)	0.43
*Gemmiger*	36	2.4 ± 0.4	2.1 ± 0.4	−0.3 (−1.1 to 0.6)	0.57

^1^ Values are expressed as the least square mean ± standard error (generalised linear mixed model). ^2^ Lactulose minus placebo. ^3^ CI, confidence interval. ^4^ An unclassified genus of the family Lachnospiraceae.

**Table A2:** (**A**). Transition in the percentages of the genus *Bifidobacterium* during lactulose ingestion ^1^. (**B**). Transition in the percentages of the genus *Bifidobacterium* during placebo ingestion ^1^.

Time of Sampling (A)	*n* ^2^	Percentage of Genus *Bifidobacterium*	*p*-Value vs. before Lactulose Ingestion
before lactulose ingestion	35	18.1 ± 11.9	-
One day after starting lactulose ingestion	27	18.0 ± 15.2	0.79
Two days after starting lactulose ingestion	28	21.5 ± 15.6	0.0044
Three days after starting lactulose ingestion	27	23.2 ± 15.1	0.0002
Four days after starting lactulose ingestion	27	22.8 ± 12.9	0.0042
One week after starting lactulose ingestion	35	25.2 ± 13.2	<0.0001
Two weeks after starting lactulose ingestion	35	27.3 ± 14.8	<0.0001
**Time of Sampling (B)**	***n*** **^2^**	**Percentage of Genus *Bifidobacterium***	***p*** **-Value vs. before** **Placebo Ingestion**
before placebo ingestion	35	16.8 ± 12.1	-
One day after starting placebo ingestion	28	15.2 ± 10.8	0.99
Two days after starting placebo ingestion	29	16.1 ± 11.3	0.76
Three days after starting placebo ingestion	30	16.4 ± 13.5	0.72
Four days after starting placebo ingestion	25	14.6 ± 10.0	0.20
One week after starting placebo ingestion	34	18.1 ± 13.2	0.58
Two weeks after starting placebo ingestion	35	17.3 ± 15.1	0.75

^1^ Values are expressed as the mean ± standard deviation (paired *t*-test). ^2^ The paired *t*-test was performed except for missing data (not all participants defaecated every day).

**Table A3:** (**A**). Transition in the cell numbers of the genus *Bifidobacterium* during lactulose ingestion ^1^. (**B**). Transition in the cell numbers of the genus *Bifidobacterium* during placebo ingestion ^1^.

Time of Sampling (A)	*n* ^2^	Cell Number of Genus *Bifidobacterium* (log CFU ^3^/g faeces)	*p*-Value vs. before Lactulose Ingestion
before lactulose ingestion	35	10.33 ± 0.93	-
One day after starting lactulose ingestion	27	10.24 ± 1.08	0.68
Two days after starting lactulose ingestion	28	10.44 ± 1.10	0.0093
Three days after starting lactulose ingestion	27	10.48 ± 1.05	0.025
Four days after starting lactulose ingestion	27	10.63 ± 0.87	<0.0001
One week after starting lactulose ingestion	35	10.64 ± 1.04	0.0025
Two weeks after starting lactulose ingestion	35	10.63 ± 0.95	0.0015
**Time of Sampling (B)**	***n*** **^2^**	**Cell Number of Genus *Bifidobacterium*** **(log CFU ^3^/g faeces)**	***p*** **-Value vs. before** **Placebo Ingestion**
before placebo ingestion	35	10.21 ± 0.76	-
One day after starting placebo ingestion	28	10.18 ± 0.97	0.65
Two days after starting placebo ingestion	29	10.26 ± 0.98	0.25
Three days after starting placebo ingestion	30	10.19 ± 1.04	0.75
Four days after starting placebo ingestion	25	10.21 ± 1.07	0.87
One week after starting placebo ingestion	34	10.31 ± 0.93	0.36
Two weeks after starting placebo ingestion	35	10.22 ± 0.95	0.93

^1^ Values are expressed as the mean ± standard deviation (paired *t*-test). ^2^ The paired *t*-test was performed, except for missing data (not all participants defaecated every day). ^3^ CFU, colony-forming units.
